# 双肺粟粒样转移的肺腺癌1例报道并文献复习

**DOI:** 10.3779/j.issn.1009-3419.2019.12.11

**Published:** 2019-12-20

**Authors:** 琳雪 李, 亮 周, 建勇 张

**Affiliations:** 563000 遵义，贵州遵义医科大学附属医院呼吸与危重症医学科呼吸二病区 The Second Department of Pulmonary and Critical Care Medicine, Affliated Hospital of Zunyi Medical University, Zunyi 563000, China

**Keywords:** *EGFR*突变, 肺粟粒样转移, 肺肿瘤, 肺腺癌, *EGFR* mutations, Lung miliary metastases, Lung neoplasms, Lung adenocarcinoma

## Abstract

**背景与目的:**

以双肺粟粒样转移为表现的肺腺癌临床表现特殊，极易误诊。本研究探讨双肺粟粒样转移的肺腺癌的临床特征，以提高临床医师对该病的认识。

**方法:**

回顾性分析遵义医科大学附属医院收治的1例经病理确诊的双肺粟粒样转移的肺腺癌患者，以“粟粒样，肺癌”、“肺癌，双肺转移”和“肺癌，结节”为检索词检索万方数据库和中国期刊网全文数据库（CNKI），以“miliary intrapulmonary carcinomatosis”、“lung cancer miliary”、“pulmonary nodule, lung cancer”和“EGFR, miliary”为检索词检索PubMed数据库，检索时间1947年1月1日-2019年5月30日。

**结果:**

患者女，52岁，因“咳嗽、咳痰2月，气促1月”于我院就诊，胸部计算机断层扫描（computed tomography, CT）提示双肺弥漫结节，经皮肺穿刺活检术后病理诊断肺腺癌、表皮生长因子受体（epidermal growth factor receptor, *EGFR*）外显子21 L858R突变。患者经口服吉非替尼治疗1个月后病情明显好转，多次复查胸部CT提示双肺结节较前减少。共检索出相关中文文献7篇，英文文献56篇，经阅读除外资料不详者及无病理诊断结果的文献，共报道16例，结合本例，对17例汇总分析。17例肺腺癌有2例未描述是否进行*EGFR*基因检测，15例*EGFR*基因检测中1例无突变，10例为19外显子缺失，1例ALK阳性，1例*EGFR*外显子21 L858R突变，2例为外显子20插入。

**结论:**

以双肺粟粒样转移改变为特征的肺腺癌临床罕见，属肺腺癌亚型，应重视其表现以避免误诊。该腺癌亚型大多有EGFR突变，EGFR酪氨酸激酶抑制剂（EGFR-tyrosine kinase inhibitors, EGFR-TKIs）是这类患者的首选治疗。

## Abstract (en)

**Background and objective:**

Lung adenocarcinoma with miliary metastasis in both lungs is easily misdiagnosed. The aim of this study is to investigate the clinical features of lung adenocarcinoma with miliary metastases in both lungs and to improve the clinician's understanding of the disease.

**Methods:**

The clinical manifestation, radiology and pathology were analyzed in one patient with miliary intrapulmonary carcinomatosis in Affiliated Hospital of Zunyi Medical University. A review of literature was performed with "miliary intrapulmonary carcinomatosis", "lung cancer miliary", "pulmonary nodule, lung cancer" and "EGFR miliary" as key words in PubMed, Wangfang datebase and CNKI.

**Results:**

The patient was a 52 year-old woman with a history of productive cough for 2 months, which aggraveted with shortness of breath for 1 month. Her computed tomography of chest showed diffuse military nodules distributed at bilateral lungs. Computed tomography (CT)-guided needle biopsy of the left lung revealed lung adenocarcinoma and epidermal growth factor receptor (*EGFR*) exon 21 L858R mutation. The patient was treated with gefitinib 250 mg per day. The chest CT was reviewed several times during this period, which shows the double lung nodules were reduced. The patient is generally in good condition and her symptoms have improved. By literature review, we found relevant 7 Chinese articles and 56 English articles, a total of 16 cases have been reported. 17 patients were lung adenocarcinoma, 2 patients did not describe whether to detect *EGFR* gene mutations, 1 patient did not have *EGFR* gene mutation; 10 patients were *EGFR* exon 19 deletion, 1 patient was ALK positive, 1 patient was *EGFR* exon 21L858R mutation, 2 patients were *EGFR* exon 20 insertion.

**Conclusion:**

Lung adenocarcinoma with miliary metastasis in both lungs is a rare phenomenon. We should pay attention to the performance to avoid misdiagnosis. Most of the adenocarcinoma subtypes have *EGFR* mutations, and EGFR-tyrosine kinase inhibitors (EGFR-TKIs) are the preferred treatment choice for this type of patients.

肺癌是全世界最为高发的恶性肿瘤^[1]^，肺腺癌是肺癌最常见的病理类型，发病率逐年升高。肺腺癌肺内转移在胸部影像上可表现为多发肺结节、胸腔积液、淋巴结肿大等，但以双肺粟粒样转移为表现者罕见，常提示血源性播散。目前国内外相关研究报道较少，现将我院收治的1例经病理确诊的双肺粟粒样转移的肺腺癌病例报道如下，并进行文献复习，总结该病临床特征，提高临床医生对该病的认识。

## 1 病例资料

患者女，52岁，因“咳嗽、咳痰2月，气促1月”于2018年3月29日入院。患者2月前感冒受凉后出现刺激性咳嗽，咳少量黄痰，1个月前出现活动后气促，未予以重视。入院体查：体温36.5 oC，脉搏106次/分，呼吸20次/分，血压135 mmHg/90 mmHg，双肺呼吸音粗，余无其他阳性体征。2018年3月28日胸部计算机断层扫描（computed tomography, CT）提示：双肺弥漫结节，考虑恶性肿瘤（肺癌并肺内转移？双肺转移瘤？）、纵隔及右肺门淋巴结增多增大；支气管镜检查提示支气管粘膜炎症改变、支气管刷检物（右B2/左B3/B6刷检）病理检查见少量异型细胞团及坏死碎片，可疑高分化腺癌；经皮肺穿刺活检术后取肺穿刺物（左肺组织）行组织病理诊断提示恶性肿瘤细胞而考虑非小细胞癌，分子病理报告为表皮生长因子受体（epidermal growth factor receptor, *EGFR*）外显子21 L858R突变；发射型计算机断层扫描（emission computed tomography, ECT）发现左股骨上段局限性放射物浓聚灶，其余骨骼未见明显骨转移征象；头颅磁共振成像（magnetic resonance imaging, MRI）示颅脑未见明显异常，上腹部平扫+增强示肝右叶包膜下小钙化灶。根据以上资料，诊断为原发性支气管肺腺癌（c-T4N0M1b双肺转移）IVa期*EGFR*外显子21 L858R（+），体能状态（performance status, PS）评分1分并肺部感染，予以吉非替尼250 mg，1次/d。治疗1个月后患者症状明显好转，2018年5月14日复查胸部CT提示双肺弥漫结节并间质性病变，与2018年3月28日胸部CT比较，双肺结节减少、缩小，纵隔及右肺门淋巴结增多增大。2018年9月擅自停服吉非替尼1个月，于2018年10月1日复查胸部CT提示较2018年5月14日胸部CT见双肺双肺弥漫结节呈明显增多、纵隔及双肺门淋巴结进一步增多、增大。考虑双肺病变加重与患者停药有关，此后患者遵医嘱一直坚持服药。2018年11月30日复查胸部CT提示双肺弥漫结节较2018年10月1日减少。院外随访至今，患者一般状况良好。

## 2 文献复习

### 2.1 文献检索

以“粟粒样肺癌”或“肺癌双肺转移”或“肺癌结节”为检索词，分别在万方数据库和中国知网期刊数据库检索，检索时间从1947年1月1日到2019年5月30日，检索出相关中文文献共7篇，报道患者2例。以“miliary intrapulmonary carcinomatosis”或“lung cancer miliary”或“pulmonary nodule lung cancer”或“EGFR miliary”为检索词，在PubMed数据库检索，检索时间从1947年1月1日到2019年5月30日，共检索出相关英文文献共56篇，报道国外病例14例。经阅读除外资料不详者及无病理诊断结果的文献，最终筛选获得16例，结合本病例，针对17例进行综合分析。

### 2.2 17例入选病例临床特点分析

#### 2.2.1 一般情况

男性8例（47.05%），女性9例（52.94%），年龄36岁-71岁，平均年龄（54.53±11.03）岁，中位年龄55岁。

#### 2.2.2 临床表现

除外5例未详细描述症状，余12例症状主要表现为咳嗽7例，咳痰2例，活动后气促8例，喘息1例，胸痛1例，呼吸困难1例，体重减轻3例。

#### 2.2.3 肺部影像学检查

胸部影像学均表现为双肺多发弥漫结节。

#### 2.2.4 病理检查

17例肺腺癌有2例未描述是否进行*EGFR*基因检测，15例*EGFR*基因检测中1例无突变，10例外显子19缺失，1例ALK阳性，1例*EGFR*外显子21 L858R突变，2例外显子20插入。

#### 2.2.5 治疗和转归

1例因病情较重，先后予以抗结核、化疗后病情恶化死亡；1例死于肺部感染，11例予以EGFR酪氨酸激酶抑制剂（EGFR-tyrosine kinase inhibitors, EGFR-TKIs）治疗后好转；2例化疗后好转，2例化疗后病情恶化死亡。具体情况见表 1。

**1:** 文献报道的16例双肺粟粒样转移的肺腺癌病例临床资料 Clinical data of 16 cases of lung adenocarcinoma with bilateral miliary metastases reported in the literature

Author	Gender	Age	Symptoms	Radiography	Pathology	Distant metastasis	Treatment	Result
Qiao YX, 2018^[2]^	Male	36 yr	Cough; shortness of breath	Cystic shadow on the left upper lobe, multiple nodules in both lungs	Lung adenocarcinoma; no *EGFR* gene mutation detected	PET/CT: diffuse nodules in both lungs	Crizotinib	Improvement
Jiang N, 2016^[3]^	Female	39 yr	Shortness of breath	Miliary pulmonary metastases	Lung adenocarcinoma; *EGFR* exon 19 deletion	Biopsy showed left cervical lymph node adenocarcinoma metastasis	Chemotherapy	Improvement
Beck TN, 2018^[4]^	Male	55 yr	Dry cough; shortness of breath	Miliary pulmonary metastases	Lung adenocarcinoma; *EGFR* exon 19 deletion	PET/CT: extensive metastasis of bilateral lung parenchyma	Afatinib	Improvement
Lim CK, 2015^[5]^	Male	64 yr	Dry cough; shortness of breath	A 2.5 cm nodule at right lower lung; miliary nodules at bilateral lungs	Lung adenocarcinoma; *EGFR* exon-19 deletion mutation	-	Afatinib	Improvement
Tejas Patil, 2018^[6]^	Female	56 yr	Shortness of breath	Innumerable and diffuse pulmonary nodules in both lungs	Lung adenocarcinoma; *EGFR* exon 19 deletion	Multiple hepatic and osseous lesions; pathologic cervical fractures at the C6 and C7 levels	Erlotinib; posterior spinal fusion and decompression	Improvement
Schaller A, 2014^[7]^	Male	47 yr	Cough; fatigue; weight loss	Miliary multiple micronodules in both lungs	Lung adenocarcinoma	-	Cisplatin-pemetrexed-bevacizumab	Improvement
Khadem N, 2017^[8]^	Male	51 yr	Palpitations; night sweats; intermittent chest pain; shortness of breath; left thigh pain; weight loss	Extensive, bilateral, randomly distributed pulmonary nodules	Lung adenocarcinoma	Left thigh	Anti-tuberculosis; chemotherapy	Death
Laack E, 2011^[9]^	Female	40 yr	-	Miliary pulmonary metastases	Lung adenocarcinoma; *EGFR* exon 19 deletion	Brain	Erlotinib	Patient was 19 months in complete remission before the tumor progressed
Laack E, 2011^[9]^	Female	70 yr	-	Miliary pulmonary metastases	Lung adenocarcinoma; *EGFR* exon 19 deletion	-	Chemotherapy; erlotinib	Improvement
Laack E, 2011^[9]^	Female	42 yr	-	Miliary pulmonary metastases	Lung adenocarcinoma; *EGFR* exon 19 deletion	-	Chemotherapy, erlotinib	4 months later, the CT scan revealed a partial remission, and the miliary lung metastases had almost disappeared
Laack E, 2011^[9]^	Male	63 yr	-	Miliary pulmonary metastases	Lung adenocarcinoma; *EGFR* exon 19 deletion	-	Chemotherapy, erlotinib	Improvement
Laack E, 2011^[9]^	Male	68 yr	-	Miliary pulmonary metastases	Lung adenocarcinoma; *EGFR* exon 19 deletion	Lymph nodes of mediastinum; malignant pleural effusions; bone metastases; one liver metastasis	Erlotinib	18 months in complete remission before the tumor progressed
Tun NM, 2015^[10]^	Female	71 yr	Dry cough and shortness of breath, significant weight loss	Innumerable bilateral opacities in a miliary pattern	Primary lung adenocarcinoma	-	Chemotherapy	Improvement
Sekine A, 2018^[11]^	Female	53 yr	Cough	Numerous miliary nodules throughout bilateral lungs	Papillary adenocarcinoma; *EGFR* exon 20 insertion	-	Chemotherapy; afatinib	Death
Sekine A, 2018^[11]^	Female	61 yr	Cough; dyspnea during exercise	Miliary lung nodules	Micropapillary adenocarcinoma; *EGFR* exon 20 insertion	-	Chemotherapy	Death
Archer G, 2019^[12]^	Male	59 yr	Dyspnea	Miliary carcinoma- tosis	Lung adenocarcinoma; *EGFR* exon 19 deletion	-	Osimertinib	Death
EGFR: epidermal growth factor receptor; PET-CT: positron emission tomography-computed tomography.								

## 3 讨论

### 3.1 肺癌粟粒样肺转移的称谓及其演变

以双肺粟粒样转移为表现的肺癌临床罕见，国内外相关报道较少。Wu等^[13]^在2013年首次将以双肺粟粒样转移表现的肺癌称为“粟粒样肺内癌”（miliary intrapulmonary carcinomatosis, MIPC），Kim等^[14]^在2015年同样以此种方式命名该病。Poonia等^[15]^在2014年报道1例*EGFR*基因外显子19缺失突变伴双肺粟粒样转移及相关中枢神经系统表现的非小细胞肺癌（non-small-cell lung cancer, NSCLC）患者，将其影像学形态表现命名为“粟粒样肺转移”（miliary metastasis）。2018年，Patil等^[6]^探讨1例*EGFR*基因外显子19缺失突变的NSCLC出现粟粒样肺转移现象，同样将其根据影像学特点命名为“NSCLC的粟粒样肺转移”（miliary metastases in NSCLC）。我国此类疾病文献报道较少，马俊德^[16]^最早于1982年提出将这类表现的疾病统称为“肺部粟粒样病变”。2018年乔一娴等^[2]^发现1例肺大泡伴双肺弥漫性结节。该病表现特殊，临床罕见，目前在国内外尚无统一的命名。

### 3.2 MIPC的影像学特征

肺癌的肺内转移在胸部影像学上有多种不同的表现，如多发肺结节、胸腔积液及淋巴结肿大等。血行转移的肺结节常为多发、直径3 mm-10 cm的结节，经血行播散而随机分布。罕见病例则以类似粟粒性结核的弥漫性微小离散结节的形式出现，结节为直径0.1 cm-1 cm的离散圆形或椭圆形病灶。MIPC的影像学判断标准为：（1）肺内多发、微小、离散、圆形结节，大小基本一致，弥散性分布于2个肺野；（2）结节数目众多在影像学上常无法确切计数；（3）肺结节多为直径≤5 mm；（4）排除单侧肺内癌或淋巴管癌患者^[14]^。

### 3.3 MIPC的病理学机制

肿瘤的影像学表现可在一定程度上反映肿瘤的分子微观特征，粟粒样肺转移的特征是数百个小转移病灶均匀分布于全肺，表明这些肿瘤病灶具有共同的分子特征。研究^[17]^证实，粟粒样肺转移与*EGFR*突变的临床相关性很高，初次诊断时有MIPC表现的NSCLC患者*EGFR*突变率高于无MIPC者。

有报道表明，15例弥散性随机肺转移的肺癌患者中有11例发生*EGFR*突变（73%），提示*EGFR*突变是肺癌全肺弥散性随机转移的主要因素。*EGFR*突变的肺腺癌患者更容易发生弥散性随机肺转移，其血源性转移的机制与血管生成有关。EGFR及其配体在肿瘤发生发展中发挥重要作用，EGFR信号通路调控肿瘤细胞中多种不同血管生长因子（如血管内皮生长因子、白介素-8和成纤维细胞生长因子）的合成和分泌，伴随肿瘤发生*EGFR*突变，容易发生血管生成性转移，如弥散、随机肺转移^[18]^。

与肺鳞状细胞癌相比，肺腺癌具有更大的肿瘤血管生成潜能，可能导致高发的血源性播散，且可能通过血源性播散发展为早期转移^[19]^。因此，腺癌是MIPC最初诊断的主要组织病理学类型。

### 3.4 MIPC的鉴别诊断

MIPC的影像学表现特殊，首先要注意与血行播散性肺结核相鉴别，本组文献复习病例中有1例因误诊为肺结核而行抗结核治疗。此外，还需注意与癌性淋巴管炎、多原发肺癌、细支气管肺泡癌、肺转移癌，结节病、煤矿工人尘肺病、矽肺病、含铁血黄素病、纤维化肺泡炎、急性外源性过敏性肺泡炎、肺嗜酸性粒细胞综合征、肺泡蛋白病、原发性甲状腺癌的血行转移及多房棘球绦虫感染所致的粟粒样肺转移表现^[20]^等疾病鉴别，组织病理是确定诊断的关键。

### 3.5 MIPC的治疗

粟粒样转移与腺癌亚型以及*EGFR*突变有关，提示EGFR-TKIs可能是这类患者的首选治疗选择。文献报道认为，MPIC大多以伴有外显子19缺失的NSCLC粟粒样肺转移为主，这些转移病灶对EGFR-TKIs有显著的应答。一项研究^[4]^报道5例从不吸烟的粟粒样转移的肺腺癌患者，*EGFR*突变基因测序均发现外显子19缺失，均对EGFR-TKIs有显著反应，推测在非吸烟者中，特别是外显子19缺失，可能是潜在的引起粟粒样转移的肺腺癌的原因。这与本组研究病例符合，9例（9/16）均为外显子19缺失，且均对EGFR-TKIs敏感。

MIPC也与包括外显子20插入突变在内的其他致癌基因驱动突变密切相关。伴有粟粒样肺转移的外显子20插入突变的NSCLC患者预后较差，进一步化疗的机会较低。外显子20插入突变的NSCLC可表现为粟粒样肺转移和疾病快速进展^[11]^。外显子20点突变仅占*EGFR*突变的4%，且与EGFR-TKIs敏感性降低有关。临床医生应警惕*EGFR*外显子20突变患者对EGFR-TKIs具有耐药性^[21]^。与第一代或第二代EGFR-TKIs相比，*EGFR*外显子20 T790M突变的患者使用奥希替尼疗效更好。外显子19缺失和外显子21突变占*EGFR*驱动突变的90%，可预测EGFR-TKIs的治疗反应。并非所有*EGFR*突变都对EGFR-TKIs治疗敏感，也不是所有有粟粒表现的患者都可能对EGFR-TKIs产生应答。对于发生粟粒样肺转移而无*EGFR*外显子19或21突变的患者，应考虑分析不太常见的驱动突变。

### 3.6 MIPC的预后

Hsu等^[19]^对543例肺癌患者分析发现，*EGFR*的突变与较高的粟粒脑和肺转移率相关，粟粒转移灶的存在并不意味着总体存活率较差，可能是因为粟粒转移与EGFR-NSCLC有利的预后亚型相关。也有学者认为粟粒播散性癌患者尽管存在*EGFR*突变激活和应用EGFR-TKIs有较好的治疗反应，但可能出现较高的疾病负担及生存不良的预后因素。如果*EGFR*突变患者对EGFR-TKIs的反应越好，预期的总生存时间就越长。

**1:**
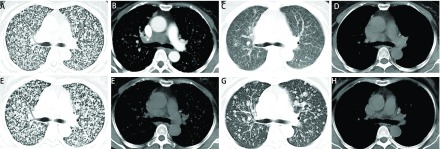
患者治疗过程中胸部影像学的变化情况。A、B：2018年3月28日胸部CT示双肺弥漫结节，纵隔及右肺门淋巴结增多、增大；C、D：2018年5月14日经吉非替尼治疗1个月后复查胸部CT示与2018年3月28日胸部CT比较，双肺结节减少、缩小；E、F：2018年10月1日再次复查胸部CT示与2018年5月14日胸部CT检查比较，双肺结节明显增多；G、H：2018年11月30日复查胸部CT示与2018年10月1日胸部CT检查相比，双肺结节减少。 Changes of chest imaging in the patient during treatment. A, B: Chest CT shows (March 28, 2018) diffuse nodules in both lungs, mediastinal and right hilar lymph nodes increased in number and size; C, D: After Gefitinib treatment for 1 month reexamination of chest CT (May 14, 2018) shows decreased lung nodules zoom out comparing with chest CT on March 28, 2018; E, F: Reexamination of chest CT (October 1, 2018) shows bilateral lung nodules were obvious increased in number comparing with chest CT on May 14, 2018; G, H: Chest CT (November 30, 2018) shows bilateral pulmonary nodules decreased comparing with chest CT on October 1, 2018. CT: computed tomography.

**2:**
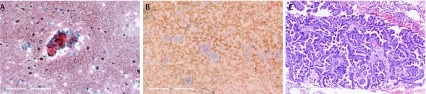
患者的HE染色病理图片。A:病理（HE染色，×200）：（支气管刷检物）少量异型细胞团及坏死碎片，可疑高分化腺癌；B：病理（HE染色，×200）：（肺穿刺物）见恶性肿瘤细胞，考虑非小细胞肺癌；C：病理（HE染色，×300）：（肺组织）肺腺癌，部分为微乳头状型。 HE staining pathological picture. A: Haematoxylin and eosin staining, 200 times magnification: (bronchial brush) a small amount of heterotypic cell mass and necrotic debris, suspected highly differentiated adenocarcinoma; B: Haematoxylin and eosin staining, 200times magnification: (lung puncture) see malignant tumor cells, considering non-small cell lung cancer; C: Haematoxylin and eosin staining, 300 times magnification:(lung tissue) lung adenocarcinoma, partially micro-papillary.

女性、不吸烟者、腺癌患者和东亚人患者对EGFR-TKIs治疗反应较好，而初次诊断为MIPC的男性和吸烟患者*EGFR*突变率也很高。MIPC改变有助于识别极有可能是*EGFR*突变的患者，在识别患者谁更可能携带*EGFR*突变，粟粒样肺转移是一种可靠的特征，当基因分析不可用或无法诊断时，这可能对我们选择EGFR-TKIs的治疗决策有用，EGFR-TKIs可能是初次诊断为MIPC的亚洲人患者（无论性别或吸烟状况）首选治疗方法^[4]^。

MIPC是一种侵袭性较强的疾病状态，大多数初诊MIPC的NSCLC患者往往病情较重^[22]^，病程迅速进展而死亡，通常不推荐化疗^[23]^。高*EGFR*外显子19突变率导致MIPC患者在初始诊断时*EGFR*突变率高于无MIPC患者。

综上所述，MIPC是肺腺癌一种罕见特殊表现形式，临床医生应提高对该病的认识，避免漏诊、误诊。MIPC的出现与*EGFR*突变关系密切，EGFR-TKIs药物可能是初次诊断为MIPC的肺腺癌患者的首选治疗方法^[13]^。
